# Utilizing rapid diagnostic tests in infectious disease surveillance and response in humanitarian emergencies

**DOI:** 10.3389/fpubh.2026.1849490

**Published:** 2026-08-11

**Authors:** Meghan E. Herilla, David A. Townes, Andrew T. Boyd

**Affiliations:** 1 Division of Global Health Protection, Global Health Center, Centers for Disease Control and Prevention, Atlanta, GA, United States; 2 Department of Emergency Medicine, University of Washington School of Medicine, Seattle, WA, United States; 3 Department of Global Health, University of Washington School of Public Health, Seattle, WA, United States

**Keywords:** conflict, humanitarian emergencies, infectious disease, point of care, rapid diagnostic tests

## Abstract

**Introduction:**

As of 2024, approximately 2 billion people live in conflict-affected regions worldwide, with an estimated 108 million forcibly displaced. Populations affected by humanitarian emergencies (HEs) are at increased risk of outbreaks of epidemic-prone diseases and significant morbidity and mortality. In these settings, access to laboratory diagnostic capabilities may be limited or challenging. Rapid diagnostic tests (RDTs) represent an essential modality for ensuring timely diagnostic testing to differentiate among infectious diseases and respond with appropriate treatment in HEs. A general landscape review of RDTs for operational use for infectious disease surveillance and response in these settings is warranted.

**Content:**

We reviewed white and gray literature to explore the availability, technical characteristics, and operational strengths and weaknesses of RDTs available for the diagnosis of selected epidemic-prone infectious diseases in HEs. We documented planned development of RDTs. Finally, we reflect on how barriers to more widespread use of RDTs in HEs could be addressed, along with potential complementary use of other near point-of-care testing, to guide infectious disease diagnosis in HEs.

**Discussion:**

Low-cost, easy to use, and accurate RDTs for epidemic-prone diseases are an integral component of infectious disease surveillance, outbreak detection, and response efforts in HEs. Field evaluations specifically in HEs, continued work to attain WHO pre-qualification, and country-level planning on operational use of RDTs are important to increasing the use of RDTs for infectious diseases in HEs.

## 1 Introduction

A humanitarian emergency (HE) is defined as “a disaster requiring international support to meet the basic needs of the affected population” (1). A subset of HEs is a complex humanitarian emergency, a “humanitarian crisis which occurs in a country, region, or society where there is a total or considerable breakdown of authority resulting from civil conflict and/or foreign aggression [and] requires an international response which goes beyond the mandate or capacity of any single agency” (2). In 2024, the Director of the Department of Noncommunicable Diseases at the World Health Organization (WHO) stated that 2 billion people live in conflict-affected regions worldwide, with an estimated 108 million either displaced internally or forced to flee their home country (3). HEs often occur in areas with weak public health infrastructure and may result in a breakdown of health services, including clinical and laboratory facilities, where equipment and materials may be degraded or damaged, supply chains disrupted, and expert personnel are displaced or killed. This breakdown of health services is often coupled with poor access to safe shelter, food, water, and sanitation, exacerbating the public health crises and creating potential for increased incidence of infectious diseases in these displaced populations (4). Infectious disease outbreaks in these settings may be difficult to detect or stop because of degradation or loss of routine public health surveillance and reporting systems.

To guide response to the increased risk for outbreaks of epidemic-prone infectious diseases in HEs, the WHO has developed guidelines for Early Warning Alert and Response (EWAR) in emergencies (5). The EWAR surveillance system is operationalized in HEs when national disease surveillance systems may not be functional and operates using syndromic surveillance, designed to utilize sensitive case definitions to generate early detection and reporting alerts of potential outbreaks. Thereafter, EWARN participants investigate these alerts to determine whether an alert reflects an outbreak. Laboratory diagnostic testing of a particular pathogen facilitates that determination (6). However, laboratory-based testing is often not feasible in HEs, as transport of samples may be disrupted, or local laboratories, equipment, or reagents may be damaged or unavailable (7). Rapid diagnostic tests (RDTs) are “simple to operate, quick testing tools that can be used directly at [the point-of-care] level, eliminating the need for advanced laboratory infrastructure or costly equipment” (8). Historically, the most common type of RDT used in emergencies is a lateral flow, immunochromatographic assay. The use of RDTs, or related “field-based” diagnostic modalities that can provide or guide initial, operational diagnosis but do not require a full laboratory, can decrease equipment, personnel, logistics, or transport requirements for laboratory specimen transfer, management, testing, and result, and thus significantly decrease the time lag for initial public health response to infectious disease outbreaks, even while gold-standard laboratory-based confirmation is pursued concurrently.

Despite the utility of RDTs for infectious disease operational diagnosis and surveillance in humanitarian emergencies, their operational use has historically been limited. The number of RDTs that are commercially available for key diseases in HEs is low compared to traditional laboratory testing methods, and some available RDTs have sensitivity and specificity too low to be considered useful. There have also been concerns about the skill of users to make decisions based on test results, and many Ministries of Health and humanitarian organizations may be wary of procuring any commercially available RDT that lacks WHO prequalification, the process by which WHO assesses the quality, safety, and efficacy of medical products. Yet with ongoing technical advancements in RDTs, a more general landscape review of RDTs for operational use in infectious disease diagnosis and surveillance in these settings is warranted.

This review updates and builds upon a 2020 paper to explore the availability, technical characteristics, and operational strengths and weaknesses of RDTs available for the diagnosis and surveillance of selected epidemic-prone infectious diseases in HEs (7). In general, we framed our points of description for available RDTs in comparison with WHO target product profiles for RDTs desired performance requirements, including a clinical sensitivity and specificity of greater than 95 and 98% respectively, produce a result in less than 30 min, require no cold chain and no additional equipment, have a shelf life of at least 24 months, and cost less than $1 USD per test ($5 USD for more logistically complex tests such as for meningitis) (9–11). We document planned development of new RDTs. Finally, we reflect on how barriers to more widespread use of RDTs in HEs could be addressed, along with potential complementary use of other near point-of-care testing, to guide infectious disease diagnosis in HEs. For the purposes of this review, we focus on RDTs for selected priority diseases corresponding to syndromes often included in EWAR systems in emergencies: measles; polio (corresponding to acute flaccid paralysis syndrome); Ebola virus disease (EVD), yellow fever, and dengue fever (corresponding to hemorrhagic fever syndrome); typhoid fever, shigellosis, enterotoxigenic *E. coli* enteritis, and cholera (diarrheal diseases); hepatitis A and hepatitis E (acute jaundice syndrome); and *N. meningitidis* meningitis (meningitis syndrome). These diseases are frequent causes in emergencies of key syndromes suggested as reportable indicators in EWAR in emergencies (6); they tend to cause outbreaks, cause high morbidity or mortality, and when identified, can be treated in an infected person, or transmission can be mitigated or prevented. Malaria is often included in the priority diseases list as well, but we chose not to include an update on RDTs for malaria, as commercial RDTs for malaria are already widely used in HEs.

## 2 Content

### 2.1 Methods

To conduct this review, we adapted the general framework of a landscape analysis to compare available RDT products (12). The types of documents considered in this review included reports from multinational, bilateral, and non-governmental organizations, news articles, and traditional peer-reviewed journals. Comprehensive literature searches were conducted using three main categories of relevant search terms: context (humanitarian, emergency, disaster), intervention (rapid diagnostic test, point-of-care test), and disease. Initial searches combined disease-specific terms with diagnostic modalities [e.g., *(disease) AND rapid diagnostic test OR point-of-care test*]. When specific diagnostic tests were identified, secondary searches were performed using the test name or disease in combination with the terms *emergency, humanitarian, or disaster* to assess evidence of operational use in resource-limited or emergency settings. We did not specifically limit searches to a particular RDT type or platform. Key databases consulted were WHO model list of essential in vitro diagnostics and WHO list of prequalified in vitro diagnostic products (13, 14). Searches were completed between October 2024 and April 2026, with sources limited to those published in English. We focused on availability, technical characteristics, and operational characteristics of RDTs for the diagnosis of selected epidemic-prone infectious diseases in HEs. Summaries of key elements of availability are listed in Table 1.

**Table 1:** List of selected epidemic-prone diseases in humanitarian emergencies and aligned rapid diagnostic tests as of April 2026.

Disease	Included on WHO essential diagnostics list (specific assay format) (13)	WHO Pre-qualification of an RDT (14)	RDT commercially available	RDT in commercial development or testing (if RDT not already commercially available)
Measles	YES, immunoassay and nucleic acid test (NAT), for use in laboratories	NO	YES	n/a
Polio	NO	NO	NO	NO^1^
Ebola virus disease	NO	NO	YES	n/a
Yellow fever	NO	NO, but WHO endorses its use for surveillance (13)	YES	n/a
Dengue fever	YES, immunoassay, NAT, RDT for use in laboratories	NO	YES	n/a
Typhoid fever	NO	NO	YES	n/a
Shigellosis	NO	NO	NO	YES
Enterotoxigenic *E. coli* enteritis	NO	NO	NO	YES
Cholera	YES, RDT, for use in communities or facilities without laboratories, for initial detection of an outbreak	NO, but Global Task Force for Cholera Control endorses for surveillance (68)	YES	n/a
Hepatitis A	NO	NO	NO	YES
Hepatitis E	YES, immunoassay, NAT, RDT for use in laboratories, and RDT for use in communities or facilities without laboratories to aid in diagnosis and surveillance of infection (conditional)	NO	YES	n/a
*N. meningitidis* meningitis	NO	NO	YES, but requires specially trained staff to collect the sample	n/a

^1^An RDT for primary immunodeficiency diseases (PID) (specifically useful to identify those with prolonged poliovirus shedding post-live vaccine) is in commercial development.

### 2.2 Measles

Measles is highly transmissible and causes significant morbidity and mortality, especially in young children, in HEs. Detection of IgM antibodies in serum can provide presumptive evidence of infection, while detection of measles RNA using rt-PCR provides laboratory confirmation (15). Commercially available RDTs to detect measles antibodies exist, but there is ongoing work to evaluate the performance of products, and there is not yet a WHO pre-qualified RDT. A promising lateral flow immunochromatographic RDT was developed in 2011 to detect measles specific IgM antibodies in both serum and oral fluid specimens. This test was evaluated using specimens collected through measles surveillance programs in Ethiopia, Malaysia, Russia (16), and Zimbabwe (17). In the first study, serum specimens resulted in a sensitivity and specificity of 90.8 and 93.6% respectively, and 90.0 and 96.2% with oral fluids. In the second study, sensitivity and specificity with oral fluid samples were 75 and 96.2%, respectively (16). This difference in sensitivity between the two studies may have been due to cases in which IgM levels were low in serum, resulting in specific antibodies not being detected (17).

The prototype RDT has also been tested as a cassette-housed device in clinical trials in Brazil, as well as unpublished field trials in India and Uganda (18). The test again showed high sensitivity and specificity (95 and 98% respectively) and was received well by surveillance staff following a two-day training program.

The test has been manufactured in small batches at about $4.50 USD each, and it is estimated that commercial production would significantly decrease costs. When using a blood sample, administering the test requires minimal training, is stable up to 40 degrees C, and has a shelf life greater than 1 year. The test requires minimal additional equipment and would be delivered with all required supplies (18). With support from the Bill & Melinda Gates Foundation and Médecins Sans Frontières, this test is under consideration for commercial development with both UK-based manufacturer Global Access Diagnostics and the Senegal-based production platform Diatropix (19, 20). Currently, the Global Measles and Rubella Laboratory Network is conducting performance evaluations of this and other commercially available measles RDTs (21).

### 2.3 Acute flaccid paralysis syndrome

#### 2.3.1 Polio

Polio contributes to morbidity, especially among children, in HEs, and outbreaks occur in under-vaccinated populations (22). Definitive diagnosis is done through isolation of virus in cell culture or by detecting the virus by RT-PCR (23). No RDT currently exists for the diagnosis of polio, and to our knowledge, there is no RDT being evaluated for diagnosis of polio or for WHO pre-qualification. An important part of polio eradication, however, is the identification of people with primary immunodeficiency diseases (PID), as they are much more likely to continuously shed poliovirus acquired from the oral polio vaccine. All existing identification techniques for individuals with PID are laboratory-based and therefore not easily accessible in low-resource humanitarian settings. The nonprofit global health organization PATH is working to advance an RDT for PID specifically for use in limited-resource settings, and three prototype lateral flow RDTs are currently being evaluated (24).

### 2.4 Hemorrhagic fever syndrome

#### 2.4.1 Ebola virus disease

Outbreaks of EVD cause high mortality, and may propagate in HEs where population displacement and insecurity lead to delays in detection and laboratory confirmation. The gold standard for diagnosis of EVD is rt-PCR testing (25). Multiple commercially available RDTs exist for the diagnosis of Ebola virus disease, including lateral flow assays and “rapid PCR” devices that use a self-contained cartridge system to simplify laboratory-based rt-PCR testing (26, 27). None has WHO pre-qualification.

In a 2023 systematic review, the authors evaluated the accuracy of RDTs, including both lateral flow assays and PCR-based tests, for diagnosing EVD. Sensitivity of lateral flow tests ranged from 62% (95% CI 53–71%) to 100% (95% CI 97–100%), and PCR tests ranged from 88% (95% CI 81–93%) to 100%. Overall, lateral flow tests had a summary sensitivity and specificity of 86% (95% CI 86–86.2%) and 97% (95% CI 96.1–97.9%), respectively, and PCR-based RDTs had a summary sensitivity and specificity of 96.2% (95% CI 92.4–98.1%) and 96.8% (95% CI 95.3–97.9%), respectively (26). It is worth noting, however, that the reported sensitivity of an RDT is influenced by its limit of detection: that is, the range of RT-qPCR CT values that the manufacturer used when evaluating product sensitivity. A recent independent laboratory evaluation of five Ebola virus RDTs found that the sensitivity for each was lower than that reported by the manufacturer when testing samples across the range of clinically relevant CT values (28).

#### 2.4.2 Yellow fever

Outbreaks of yellow fever cause high mortality and can propagate in endemic countries, several of whom face ongoing HEs. Definitive laboratory diagnosis generally requires detecting yellow fever virus-specific IgM and neutralizing antibodies; positive results are confirmed with a more specific test, such as a plaque-reduction neutralization test. Testing may also include virus isolation or viral RNA detection by rt-PCR, but transient viremia means negative viral isolation or rt-PCR does not rule out the diagnosis (29).

The Standard Q Yellow Fever IgM test kit is a commercially available immunochromatographic test for the rapid presumptive diagnosis of yellow fever for use in surveillance. This test was evaluated in Ghana using both serum and plasma samples, with a resulting sensitivity of 96.3% and specificity of 97.9% (30). This test was also evaluated for the WHO in the context of surveillance using serum samples, and the results agreed with the findings of the study in Ghana. Though this test has not received WHO pre-qualification, WHO has determined that this test is a useful tool for detection of yellow fever and recommended its use in the context of surveillance in 2023 (31).

The Standard Q Yellow Fever IgM test can provide a result in 15–20 min and does not require cold chain, with a storage temperature of 2–40 degrees Celsius. The handling of this test does not require specific training and is suitable for point-of-care testing. This test has a shelf life of 24 months (32).

#### 2.4.3 Dengue fever

Outbreaks of dengue fever contribute to morbidity in HEs in endemic countries. Laboratory confirmation of acute dengue virus infection requires a positive nucleic acid amplification test (NAAT, e.g., rt-PCR) or non-structural 1 (NS1) antigen test (33). Several dengue RDTs are commercially available for detection of acute dengue virus infections and are used in low-resource settings (34, 35). However, these tests are available to purchase with little regulation and with minimal or variable information on test performance (36). There is no WHO pre-qualified RDT for dengue fever.

One commercially available RDT, the SD BIOLINE RDT, which detects NS1 antigen and dengue fever virus specific antibodies, has shown mixed results in evaluations of accuracy (37). In two separate studies in Brazil and Malaysia, combined sensitivity and specificity were 46.8 and 94.4% and 82.4 and 87.4%, respectively (37, 38). In a study in French Guiana, sensitivity and specificity of the RDT NS1 antigen assay alone, compared with rt-PCR, were 87 and 92%, respectively (39). It was also noted by lab technicians that the interpretation of results was difficult because of the repeated occurrence of faint positive bands; however, this was mitigated with technician training (39).

### 2.5 Diarrheal disease

#### 2.5.1 Typhoid fever

Typhoid fever outbreaks are common in HEs, where population displacement and crowding along with inadequate water, sanitation, and hygiene (WASH) conditions are present. Definitive diagnosis is made by blood culture (40, 41). The most widely used test for the diagnosis of typhoid is the Widal test, a serological diagnostic developed in the late 1800’s. This is an agglutination test detecting *Salmonella* antibodies, performed using serum samples, and is routinely used in LMICs with a high prevalence of typhoid (42). However, the sensitivity and specificity of this test are poor. In one study testing febrile patients in Nepal, the sensitivity and specificity of the Widal test were 72 and 58% respectively, and the positive predictive value (PPV) was only 38% (43). Another study in Bangladesh produced similar results, with a PPV of only 25% (44).

Several commercial diagnostic RDTs for typhoid fever exist, but rigorous reviews have found that none have sufficient sensitivity and specificity (45–47). There is no WHO prequalified RDT for typhoid fever. In fact, an application was submitted to the WHO Strategic Advisory Group of Experts on In vitro Diagnostics (IVDs) for their 2022 meeting to list typhoid RDTs as “Do Not Do” in the WHO Essential Diagnostics List (the evidence-based register of IVDs maintained by WHO to guide countries on improving access to diagnostics), but the group determined that this would be too strong of a step as typhoid testing is a high priority area. They did acknowledge that typhoid testing requires better data (13).

Recent developments hold promise for improving this landscape in the future. A prototype test, Chembio Dual Path Platform Typhoid assay, is an immunochromatographic point-of-care assay, consisting of a test cassette and a portable, battery-powered reader instrument, which detects IgA responses to two *Salmonella typhi* antigens, A 2024 laboratory evaluation of this test found a sensitivity and specificity using manufacturer’s thresholds of 97.8% (95% CI 94.6–99.2) and 65.3% (95% CI 58.5–71.6), and at Youden’s optimal threshold, overall sensitivity of 89.7% and specificity of 82.2% (48). The manufacturer has received a grant to further develop this test (49).

#### 2.5.2 Shigellosis

Shigellosis causes significant morbidity among children under five in settings with poor WASH conditions, like HEs (50). Confirmatory diagnosis is by stool culture (51). There is no WHO prequalified RDT. A dipstick RDT was developed in 2007 by Institut Pasteur to diagnose the major causative pathogens of shigellosis. It is a dipstick test based on an immunochromatography technique used with a stool sample, rather than using a bacteria culture, and has been evaluated in Vietnam, Senegal, Chile, and Bangladesh (52).

In Vietnam, the specificity and sensitivity of the dipstick RDT for *Shigella flexneri 2a* were 99.2 and 91.5%, respectively (53). In Chile, Vietnam, India, and France, specificity was 96% and sensitivity was 100% when testing for *Shigella sonnei*. Testing for *Shigella dysenteriae* 1 in India, Vietnam, Senegal, France, and DRC resulted in a specificity of 98.7% and a sensitivity or 91.7% (54). The overall sensitivity and specificity across 10 studies covering 4,056 tests were 98 and 97%, respectively (55). The test provides results within 5–15 min, can be stored and transported at room temperature, and is provided in individual waterproof bags to avoid moistening of the dipsticks due to humidity. It was last reported in 2017 that commercialization of this test was in progress, but no more recent updates on the market availability were available in online documents (54).

#### 2.5.3 Enterotoxigenic *Escherichia coli* enteritis

Enterotoxigenic *Escherichia coli* (ETEC) is a very common cause of diarrheal disease, and a major cause of infant mortality in settings with poor WASH conditions (56). Confirmatory diagnosis of *E. coli* is by stool culture, although specific diagnosis of the ETEC pathotype requires molecular testing (57). There is no WHO pre-qualified RDT.

An RDT to detect both ETEC and *Shigella* in stool or blood samples using loop-mediated isothermal amplification platform (LAMP) was developed by researchers at Johns Hopkins University. Known as the Rapid LAMP-based Diagnostic Test, or RLDT, this test was developed for use in low- and middle-income countries and is still being evaluated; it is not yet commercially available. When tested using stool samples from children under 5 with diarrhea in India, sensitivity and specificity for Shigella were 93 and 100%, and for ETEC were 98 and 97% (58).

The RLDT is cold chain and electricity-free; required equipment for sample preparation comes in a kit, though a separate battery-powered heat block or water bath is required, and a specific battery-powered fluorometer reader is recommended for running the test. ETEC and *Shigella* can be detected directly from the stool with results in less than 1 h (59).

#### 2.5.4 Cholera

Cholera is a major contributor to morbidity and mortality in HEs, with high numbers of outbreaks in countries experiencing HEs and with case fatality rates higher than in non-HE settings (60). Laboratory confirmation is through stool culture including seroagglutination or PCR (61). There are several commercially available RDTs for cholera, with Crystal VC the most frequently used, according to a 2021 meta-analysis of field studies and technical guidance. In this meta-analysis, evaluation of the accuracy of five cholera RDTs yielded a pooled sensitivity of 91% (95% CI 87–94%) and specificity of 80% (95% CI 74–84%), respectively. Using direct testing, Crystal VC had a pooled sensitivity and specificity of 91% (95% CI 86–95%) and 75% (95% CI 69–81%) respectively, while Cholkit had a pooled sensitivity and specificity of 89% (95% CI 74–96%) and 87% (95% CI 84–90%), respectively (62).

The Crystal VC RDT can detect both the O1 and O139 serotypes of cholera, but the bivalence of this and other RDTs has led to issues with sensitivity and specificity. Crystal VC also produces a test specifically to detect the O1 serotype, which has proved to be more sensitive and specific. In the first field evaluation of the Crystal VC-O1 dipstick, sensitivity was 97.5% and specificity was 100% (63). In another evaluation, the sensitivity and specificity were 90 and 94%, respectively (64). Cholera RDTs have also shown increases in sensitivity and specificity with an enrichment step added, which involves inoculating rectal swabs with alkaline peptone water. In one study, the enriched RDT had a sensitivity of 99% and a specificity of 90% (65). In another, the enriched RDT had a sensitivity of 86.1% and a specificity of 100%. It has been recommended and is now included in the package insert of the Crystal VC test to perform the initial screening on direct stool with a “confirmation test” on enriched samples 4 h later, though operationally this time delay makes the test no longer rapid (66).

WHO has a specific target profile for a cholera RDT: either a dipstick or cassette with a sensitivity of 90% and specificity of 95%, produce results within 30 min, operate between 10 and 40 °C, cost less than $5 USD, and have a shelf life of at least 12 months with no cold chain required (9). The Crystal VC test falls within almost all of these requirements at a cost of $1.90 USD and no cold chain required, but the specificity of 75% is lower than the target (67). The limitations in accuracy in current cholera RDTs drive the recommendation from the Global Task Force on Cholera Control that cholera RDTs not be used for individual confirmatory diagnosis; however, they still have operational utility in surveillance and outbreak detection in HEs (62, 68).

### 2.6 Acute jaundice syndrome

#### 2.6.1 Hepatitis A

Hepatitis A causes disease outbreaks and high morbidity in settings with poor WASH conditions. Confirmatory diagnosis is through detection of anti-hepatitis A (HAV) IgM antibodies in the blood, or through rt-PCR to detect hepatitis A RNA. There is no WHO-prequalified RDT (69, 70).

As of November 2023, there are no commercially available RDTs for detection of anti-hepatitis A virus (HAV) antibodies, though several are being evaluated (71). The most promising test seems to be the EuDx-HE (A, B, C) kit, an immunochromatographic test that produces results for the presence of IgM anti-HAV, along with hepatitis B surface antigen and IgG anti-hepatitis C virus, within 15 min. In tests on serum samples, the kit showed acceptable clinical performance for detecting IgM anti-HAV, with a sensitivity and specificity of over 90% (72).

There is also no commercial point-of-care test available for detection of HAV RNA. An RT-LAMP assay and a recombinase polymerase amplification (RPA) assay do exist and may be useful in certain low resource settings, but both use laboratory-based procedures. However, it may be plausible to develop a field-based RDT through adaptations to these modalities (71).

#### 2.6.2 Hepatitis E

Hepatitis E also causes disease outbreaks in settings with poor WASH conditions and in displaced person settings, with especially high risk of mortality among infected pregnant women. Confirmatory diagnosis is through detection of anti-hepatitis E virus (HEV) IgM antibodies in the blood, or through rt-PCR to detect hepatitis E RNA (13, 73, 74). As of November 2023, there are commercial point-of-care tests available for Hepatitis E antibodies, but none of them have been approved by the FDA or prequalified by the WHO (71). These include the Assure HEV IgM rapid test, with 92.6% sensitivity and 100% specificity, and the Wantai HEV IgM Rapid Test, with 96.1% sensitivity and 100% specificity (75).

The Assure kit should be stored between 2-28^o^ C and can be used with a serum, plasma, or whole blood sample. This is a cassette test with very straightforward results interpretation (76). The Wantai test is also a cassette and works with serum and plasma samples. The test can be stored at room temperature with an 18-month shelf life, all accessories are provided, and results can be read within 10 min (77).

Of note, the WHO Strategic Advisory Group of Experts on IVDs, balancing utility of HEV IgM antibody RDTs for early outbreak detection in settings without robust access to confirmatory diagnostic methodologies with the need for more robust test performance data, conditionally listed the HEV IgM antibody RDT category for use in community settings and facilities without laboratories to aid in diagnosis and surveillance of HEV infection in the WHO essential diagnostics list after their 2022 meeting (13).

### 2.7 Meningitis syndrome

#### 2.7.1 *Neisseria menigitidis* meningitis

*Neisseria meningitidis* meningitis causes outbreaks in HEs, with high morbidity and mortality, especially in settings with population overcrowding and malnutrition. Confirmatory diagnosis is through cerebrospinal fluid (CSF) culture. There is no WHO-prequalified RDT (78).

A 2020 review cataloged the commercially available RDTs for diagnosis of *N. meningitidis* meningitis, using different modalities. The single commercially available immunochromatographic test (Biospeedia Meningospeed) can be stored at room temperature and can be read within 15 min (79). It has a sensitivity and specificity of greater than 90%. Its limitations include its inability to detect serogroup B, and the cost per test (as of 2020) is 27 USD, still rather expensive. Importantly, a major operational limitation is that the RDT, like the gold standard diagnostic test, requires a CSF sample; obtaining this CSF sample can only be accomplished through a lumbar puncture, which must be performed by specially trained staff, making its operational use challenging in HEs (80).

The three available latex agglutination tests (LATs) produce results within 20 min, but they require preparing specimens by boiling and centrifugation and are generally more costly than immunochromatographic tests. They also require cold storage and must be operated by experienced staff. There are several molecular tests as well, including real-time polymerase chain reaction (rt-PCR) tests and a LAMP-based platform. The rt-PCR tests have high sensitivity, but those requiring nucleic acid extraction first are labor-intensive, all are expensive, and running these tests requires purchasing and maintaining PCR instruments, as well as sufficient space for unilateral workflows and electricity to run them (80). The Eazyplex CSF direct platform, the LAMP platform, uses an instrument that is battery-operated, but this represents an additional one-time fixed cost. It also requires a CSF sample.

## 3 Discussion

As this review illustrates, the availability and utility of RDTs to guide diagnosis of priority diseases for EWAR in HEs vary by the disease. Some have no commercially available RDT (polio, shigellosis, ETEC, hepatitis A). Some have a commercially available RDT, but ongoing performance evaluations have precluded wide use (measles), poor sensitivity renders them not useful for decision-making (typhoid fever, dengue fever) or limits their use to surveillance (yellow fever, cholera, hepatitis E) or as an ancillary “rule-in” tool (Ebola virus disease), or the RDT is accurate but there are other operational limitations to its use in HEs (*N. meningitidis* meningitis). Reflecting on these varied barriers may facilitate devising solutions to improve the use of RDTs in HEs.

For those priority diseases for which commercial RDTs already exist, these RDTs could be a useful resource in humanitarian health response, especially in areas where these diseases are endemic. Many of these, however, have various technical limitations in meeting WHO target product profiles for RDTs, especially lower sensitivity and specificity than desired targets, and operational limitations for use in these settings, like high cost, cold chain storage requirements, and short shelf lives. One strategy, then, to increase use of RDTs in HEs for these priority diseases is to innovate around existing lateral flow-based tests to improve their accuracy to meet WHO target levels.

Even when RDTs are not WHO pre-qualified for disease diagnosis, or when commercially available RDTs do not meet all of the targets in the WHO target product profiles, an important use case for selected RDTs for selected diseases in HEs is for population-level disease surveillance, including for use in the “field” outside clinical laboratories. For instance, WHO endorses the Standard Q Yellow Fever IgM test for surveillance, and the Global Task Force for Cholera Control endorses use of RDTs for early detection of probable outbreaks and monitoring outbreak trends (31, 68). In addition, an evaluation of the field performance of a commercially-available hepatitis E RDT in an HE found it had higher sensitivity than parallel PCR, strengthening the evidence for utility in early detection or surveillance of outbreaks (81). For these selected conditions, RDTs can provide rapid signals of emerging outbreaks, or iterative signals of ongoing outbreak dynamics, in the disrupted conditions of HEs. These RDTs could also be helpful in efficient targeting of response interventions. With RDT use in surveillance for these diseases, it is important to also define pathways for complementary sample confirmatory testing. Use of electronic systems for recording RDT results and linking to confirmatory testing could strengthen these pathways, as would incorporating RDT use in EWAR workflows in HEs.

Other strategies to increase use of RDTs in HEs are to innovate around diagnostic modalities for these priority diseases to make field-based molecular diagnostics more feasible, or to improve links with established near point-of-care rapid rt-PCR infrastructure. While lateral flow-based tests remain the most common modality for RDTs in HEs, for many of the operational reasons listed in the WHO target product profile, operational innovations in molecular diagnostics are making their use in these settings more feasible. The LAMP-based molecular platform, which requires no cold chain or electricity, is an intriguing modality for these settings, if the fixed cost of the equipment could be brought down. Additionally, near point-of-care rapid rt-PCR modalities could be leveraged to facilitate diagnosis of selected diseases. Cepheid GeneXpert is a cartridge-based rt-PCR platform that has been used for integrated HIV and TB testing in rudimentary laboratories in low-income and rural settings (82) and for COVID-19 in humanitarian emergencies (83). It has an Ebola testing cartridge that has been used successfully for rapid diagnosis of Ebola virus disease in DRC (84). The Biofire FilmArray panel is a multiplex rapid rt-PCR platform that tests for multiple pathogens by syndrome. These rt-PCR modalities do require electricity, maintenance, training, and greater upfront and material costs, but where they are already in place in HEs, or could be rapidly transported and set up near the HE, they can be harnessed for near point-of-care rapid diagnostics for selected priority diseases.

Among the priority diseases, even if an RDT with acceptable accuracy is commercially available, none of them have WHO prequalification, a major practical barrier to their use by Ministries of Health or non-governmental humanitarian health actors in HEs. There is no universal regulatory agency for diagnostic tests, so countries without their own national regulatory agency often look to the list of WHO Prequalified in vitro Diagnostics (IVDs) to determine which diagnostic tests are safe and effective and to guide procurement decisions. However, the application and evaluation process to determine if a test should be prequalified by the WHO often spans months or years. For a product to be eligible for the list of prequalified IVDs, it must be the original product from the original manufacturer, commercially available, and of interest to UN organizations and other procurement agencies for operational use. This interest is defined by five eligibility principles: “need for IVDs for a particular disease or disease state; appropriateness of the product for use in resource-limited settings; requests from WHO Member States for particular IVDs; recommendation in WHO disease-specific testing guidelines; and availability of prequalified products that are of a similar assay format and/or assay principle” (85). Manufacturers can submit an application, and the product is then subject to dossier review, quality management system review, a product performance evaluation, and a labeling review (86). The process also requires an assessment fee of $5,000 - $12,000 USD to be charged to the manufacturer, as well as an annual fee of $4,000 USD to remain on the Prequalified IVD list (87).

As of July 2026, the WHO list of Prequalified IVDs consisted of 126 products, with only 8 infectious diseases represented: SARS-CoV-2, tuberculosis, hepatitis B, hepatitis C, HIV, HPV, malaria, and syphilis. Seventy-four of these 126 products are RDTs, listing 32 for HIV, 23 for malaria, eight for hepatitis C, two for hepatitis B, three for syphilis, and two for SARS-CoV-2, as well as three joint HIV/syphilis tests and one joint HIV/syphilis/hepatitis B test (14). When comparing the recent literature to the 2021 paper by Chadwick, Townes, and Perrone (who examined a slightly different list of priority diseases), we note a few changes: specifically, there are now no documented WHO-preapproved RDTs for cholera or dengue fever. This lack of recent changes in pre-qualification of existing RDTs for these priority conditions highlights the need for continued work to attain and maintain pre-qualification. Alternatively, it could suggest that reliance by Ministries of Health or non-governmental humanitarian health actors on WHO pre-qualification for use may be overly limiting, and that other mechanisms for reviewing RDTs in resource-challenged and emergency settings could be considered.

Beyond the practical barrier of product pre-qualification to enable the use of RDTs in HEs, remaining operational and implementation barriers exist and can be addressed. Many health practitioners and public health organizations in HEs are familiar with and use WHO pre-qualified lateral flow-based RDTs for malaria diagnosis, but may be hesitant to use an RDT with only moderate accuracy to make clinical management decisions, or may be limited in how they use them by Ministry of Health guidelines. Even if commercial RDTs are available in an HE, staff may not have test validation processes to incorporate these assays into their diagnostic workflow or the training to run or interpret the results, emphasizing the need for organizations to develop test-specific validation processes and staff proficiency testing. Additionally, humanitarian organizations may not be able to afford to stockpile RDTs for epidemic-prone diseases because of the cost and unpredictability of use, and instead may rely on stockpiling of RDTs by the Ministry of Health or WHO. Predictable use patterns of RDTs (for those priority diseases that are endemic or have seasonal patterns) or the ability to reliably and quickly purchase commercially available RDTs in an outbreak at the national or a regional level and documenting these supply chains in a humanitarian response plan, may ameliorate this issue.

Limitations to the use of RDTs for these priority diseases in HEs are numerous. Breaking down these barriers requires a multi-faceted approach. For those diseases without a commercially available RDT, further prototype testing and innovation are necessary to improve test performance and characteristics to WHO target levels. For diseases with an RDT already commercially available, there is a need to address technical limitations of the tests themselves and the functional limitations of their use in HEs, or to expand the reach of and integration of existing molecular testing platform networks. Very little of the data on field RDT performance is done in HEs, so increasing field evaluations of RDTs in HEs is important to ensure that an RDT’s field diagnostic accuracy in an HE is acceptable and that unique challenges of operational use in HEs are noted and can be addressed. It is also important to engage with Ministries of Health and humanitarian response organizations to ensure RDT use in HEs, and for which use cases, is included in national guidelines and humanitarian response plans.

Low-cost, easy to use, and accurate RDTs for epidemic-prone diseases can positively infectious disease surveillance and response efforts in HEs. Field evaluation of RDTs specifically in HEs, continued work to attain and maintain WHO pre-qualification or a practical expansion of mechanisms of approval for RDT use in HEs, and country-level planning on operational use, purchase, and storage of RDTs to facilitate outbreak detection and response are important to increase the use of RDTs in HEs.
